# Supplementary material for: Handwriting patterns in isolated rapid eye movement sleep behaviour disorder
Source: J Parkinsons Dis. 2026 Feb 18:1877718X251412236. Online ahead of print. doi: 10.1177/1877718X251412236 (PMC13347596; doi:10.1177/1877718X251412236)
Supplement: Supplementary file 1 [file sj-docx-1-pkn-10.1177_1877718X251412236.docx]

***GRAPHOLOGICAL DESCRIPTION OF EVALUATION CRITERIA FOR HANDWRITING ASPECTS***

As a graphologist I was asked to evaluate a number of handwriting samples and to make assessments based on the following aspects of handwriting features.

# MICROGRAPHIA

Micrographia being abnormally small handwriting and in particular decreasing size of writing within a given line or sample.

Each patient was given a standardised line to write “Mary had a little lamb it’s fleece was white as snow”

Individual letters were divided up into 3 separate zones:

Upper zone as in tall strokes of letter ‘f’, ‘h’,’l’ for instance

Middle zone as in those parts of individual letters that rest on illusory base line of unlined paper and especially small letters such as ‘a’, ‘e’, ‘o’ etc Lower zone as in any stroke below illusory base line such as tails of ‘y’,’g’ etc.

Measurements were made of each zone as above in first word of each sentence. Measurements were also taken of each zone in last word of each sentence (such word was not consistent in samples as authors were given free will with regard to word placement on paper).

Reduction in overall letter and zone size was noted with more than 25% decrease in any given zone (or all 3) to the mm. and this was catagorised as abnormal.

Micrographia is not a feature in non neurologically affected individuals.

# SENTENCE SLOPE

Sentence slope was judged and measured by means of a protractor.

**+**or - 10 degrees in a given line of handwriting was considered abnormal and out of range for healthy copybook.

# TREMOR

Observations and examination under microscope to look for uneven pen marks deviating from copybook continuous stroke and resultant pen shake.

Both handwriting and archimedes spirals were observed and inconsistent strokes noted as abnormal.

# RETRACING

Retracing or secondary pen marks overlying primary pen strokes can be common in healthy non abnormal writing and in particular within the formation of narrow loops in both upper and lower zone such as in letters ‘f’ and ‘y’ and ‘h’.

Retraced letter formations were assessed and noted when NOT in conjunction with formation of loops and considered micro neurological lapse and noted as abnormal.

# RESTING MARKS

Resting marks indicative of the cessation of fine motor activity is assessed as normal arising at the end of individual words or natural pen lifts within words.

Resting marks detected by heavier pressure within the pen stroke itself is considered highly indicative of unnatural neurological activity, memory loss or external interruption and is abnormal. Assuming absence of external interruption resting marks, heavier pressure resulting in spots of darker marking were assessed as abnormal.

# EXCESSIVE PEN PRESSURE

Pen pressure was evaluated as light, medium or heavy. The amount of the weight exerted from the upper limb will have an effect on pen operating skills and resultant pressure placed upon paper by writing instrument.

In original samples of handwriting pen pressure was assessed by reverse indentation made by the writing instrument on the paper. Palpable indentations categorised as excessive or for this purpose, abnormal.

Scanned copies had excessive pen pressure evaluated by darkness of pen stroke on paper.

Excessive pressure from the pen is consistent with upper body pressure. **IRREGULAR SHAPE OF LETTERS**

Shape of letter formations was evaluated in comparison to standard cursive handwriting copy book style mainly D’Nealian or Palmer method.

Irregular shapes evaluated by gross deviation from standard cursive to form distorted and deviated configurations of the above methods of copybook style and considered abnormal,

# WORD SPACING

Space between individual words were measured.

Normal spacing was assessed by comparison to the overall size (length) of any given word.

More than 30% of the overall length of the word as the space between the given words and consistent within a sample was assessed as ‘wide’.

Narrow spacing between words was assessed as less than 20% of overall length of given words and assessed as ‘narrow’.

# HANDWRITING SPEED

Handwriting speed was assessed by non graphological means although graphological assessment was also made by the assessment of:

Resting marks

Disjointed letters

Lack of fluidity of strokes

Irregularity of strokes

The above graphological features were given indicators of slow speed considered abnormal.
